# Network switching strategy for energy conservation in heterogeneous networks

**DOI:** 10.1371/journal.pone.0172318

**Published:** 2017-02-27

**Authors:** Yujae Song, Wooyeol Choi, Seungjae Baek

**Affiliations:** 1 ICT R&D Unit, Korea Institute of Ocean Science and Technology, Gyeonggi-do, Korea; 2 Ground System Development Team, Korea Aerospace Research Institute, Daejeon, Korea; Southwest University, CHINA

## Abstract

In heterogeneous networks (HetNets), the large-scale deployment of small base stations (BSs) together with traditional macro BSs is an economical and efficient solution that is employed to address the exponential growth in mobile data traffic. In dense HetNets, network switching, i.e., handovers, plays a critical role in connecting a mobile terminal (MT) to the best of all accessible networks. In the existing literature, a handover decision is made using various handover metrics such as the signal-to-noise ratio, data rate, and movement speed. However, there are few studies on handovers that focus on energy efficiency in HetNets. In this paper, we propose a handover strategy that helps to minimize energy consumption at BSs in HetNets without compromising the quality of service (QoS) of each MT. The proposed handover strategy aims to capture the effect of the stochastic behavior of handover parameters and the expected energy consumption due to handover execution when making a handover decision. To identify the validity of the proposed handover strategy, we formulate a handover problem as a constrained Markov decision process (CMDP), by which the effects of the stochastic behaviors of handover parameters and consequential handover energy consumption can be accurately reflected when making a handover decision. In the CMDP, the aim is to minimize the energy consumption to service an MT over the lifetime of its connection, and the constraint is to guarantee the QoS requirements of the MT given in terms of the transmission delay and call-dropping probability. We find an optimal policy for the CMDP using a combination of the Lagrangian method and value iteration. Simulation results verify the validity of the proposed handover strategy.

## Introduction

Recently, there has been an exponential growth in mobile traffic globally [1]. To cope with the rapid increase in the traffic, various issues and solutions have been discussed in the field of wireless communication. Heterogeneous networks (HetNets), which involve the co-deployment of multiple small base stations (BSs) such as pico and femto BSs together with traditional macro BSs, appear to be one of the economic and efficient solutions being considered [2]. The utilization of densely deployed small BSs not only offers a rich dimension for realizing increases in system capacity, but also fills coverage holes inside the initial deployment of macro BSs. However, one of the problems that arise due to a large-scale deployment of small BSs is an increase in energy consumption. This increase directly results in increased greenhouse gas emissions, which are recognized as a major threat to environment protection and sustainable development. To tackle this problem, there have been studies into a variety of techniques that can enhance the energy efficiency in HetNets. Of the techniques studied, most are based on the on/off switching of small BSs depending on the time-varying traffic load [3–5]. The work in [3] proposes a switching-on/off-based energy-saving algorithm. The main design principle of the algorithm [3] is to turn off a BS one-by-one, which will minimize the effect on the network performance by using the concept of network-impact. In [4], a dynamic small BS on/off scheme for traffic offloading is proposed, where an optimization problem is formulated as a Stackelberg game with the objective of maximizing the packet throughput with minimum energy consumption. In two-tier HetNets, the authors derive optimal BS densities that minimize the network energy consumption, while simultaneously satisfying the different delay requirements of two packet classes [5]. In addition, radio-resource allocation methods for improving network energy efficiency are proposed [6–8]. In [6], energy-efficient resource allocation for orthogonal frequency-division multiple access (OFDMA) systems is presented for parallel transmission utilizing multiple BSs. In the formulation for the optimization, the objective is to maximize the network energy efficiency while satisfying the quality of service (QoS) requirements of mobile terminals (MTs) given in terms of a minimum data rate. In [7], the authors proposed energy-efficient power allocation for cognitive radio (CR) OFDMA systems. In the formulation for the optimization, the objective is to minimize the network energy efficiency while guaranteeing the minimal data rate requirement of a secondary user in CR systems under the total power and mutual interference power constraints. The resource allocation method introduced in [8] utilizes a threshold for the macrocell radio resource occupancy to trigger the switching on/off procedure of the BS resources. Further, a load-balancing procedure is considered in order to minimize the service disruptions that may occur due to a radio resource shortage and to reduce the power consumption during the operation of HetNets. However, few existing works aim to improve the energy efficiency in HetNets through network switching, i.e., handovers. In [9], a Markov decision process (MDP) optimal policy aims to conserve the energy of BSs by optimally switching MTs between macro and small BSs. However, it does not address QoS requirements or various handover parameters. In addition, an MDP was used in [10] and [11] to formulate a handover decision-making problem in HetNets. In [10], the MDP reward function takes into account the signaling cost that is incurred when handovers are performed as well as network resources that are consumed during the connection. An optimal policy was found to maximize the expected total reward per connection. The work in [11] is an extension of [10], where the MT’s monetary budget is considered as an additional handover parameter. However, the works in [10] and [11] do not consider the energy efficiency in HetNets as a handover decision metric.

In this paper, we propose a handover strategy that helps to enhance the energy efficiency of BSs in HetNets. Our objective is to minimize the energy consumption of BSs while serving a traffic flow, while guaranteeing two MT QoS requirements, namely the transmission delay and call-dropping probability. When making a handover decision, the proposed handover strategy that is employed to achieve the goal is to reflect the effect of the stochastic behavior of handover parameters (i.e., link qualities and the MT’s mobility) and consequential energy consumption due to handover execution. This is a marked difference from existing handover studies that not only focus on current system states, but also do not consider the energy consumption generated during the handover execution. To illustrate the validity of the proposed handover strategy through an analytic exploration of the energy-saving potential for optimization in terms of the energy consumption at BSs, we formulate a handover decision problem by using a constrained MDP (CMDP). Using this, the effects of stochastic handover parameters (i.e., the link qualities and MT’s mobility) and consequential handover energy consumption may be captured when calculating the expected total energy consumption, which is the handover decision metric in this work. Moreover, in our CMDP formulation, we considered both real-time and non-real-time calls by introducing different levels of sensitivities to delay. The CMDP cannot be solved using solely tradition dynamic programming such as policy iteration or value iteration. Thus, we solve the CMDP by using a combination of the Lagrangian method and value iteration.

## System model

### Network model

**Fig 1:**
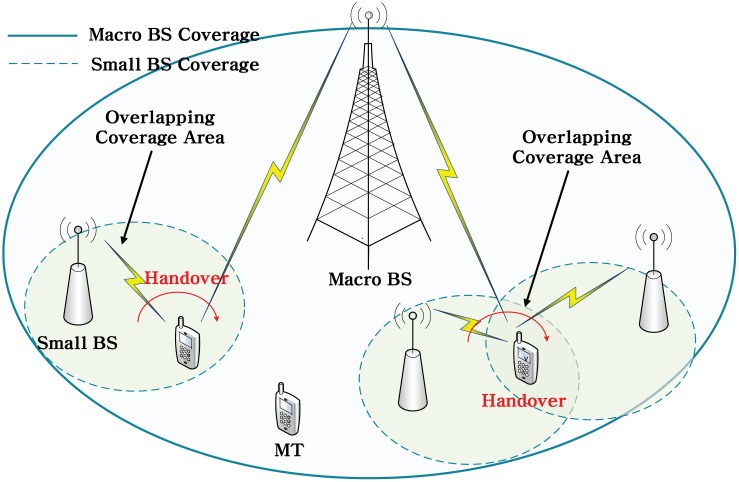
Illustration of HetNets in which a macro BS is co-located with multiple small BSs.

We consider HetNets in which multiple small BSs are located within the coverage of a macro BS, as illustrated in Fig 1. BSs belonging to different network types have different transmission powers and cell coverage, whereas BSs that belong to the same network type have the same transmission power and cell coverage. The frequency reuse factor between BSs is assumed to be unity, which implies that all BSs operate within the same spectrum, regardless of the network type. In this work, we model the two-dimensional (2D) location of MTs by using a continuous random variable with a uniform distribution, and we study the handover performance of a randomly selected MT. Let N be a set of BSs to which the MT can connect, such that N={1,2,…,|N|}, where |N| is the cardinality of the set. Of the accessible BSs, the BS to which the MT is currently connected referred to as a serving BS, and a BS that the MT considers for handover is referred as to a target BS. Handover decision making is performed at time instances that are called handover decision epochs.

### Link model

Let link *n* be a downlink wireless channel between the MT to BS n∈N. Then, the signal-to-interference-plus-noise ratio (SINR) of the MT connected to BS *n* is defined as $SINR_{n}\left(Q\right)=P_{n}Q_{n}/\sum_{j\in N,j\neq n}P_{j}Q_{j}+\sigma^{2}$, where *P*_*n*_ is the transmission power of BS *n*, $Q=[Q_{1},...,Q_{n},...,Q_{\left|N\right|}]$ is the set of link qualities between the accessible BSs and the MT, and *σ* is the additive white Gaussian noise. In the SINR definition, we assumed that the interference from BSs to which the MT cannot access is neglected because the amount of interference received from them is much smaller than that from the main interferers (i.e., N\n). In this work, the channel quality of link *n*, which is denoted by *Q*_*n*_, is assumed to be constant during the service time of a frame, and it is modeled as a finite state Markov chain (FSMC) [12]. The FSMC partitions the link quality into a finite number *M* of non-overlapping intervals, i.e., 0 = *γ*_0_ < *γ*_1_ < … < *γ*_*M*−1_ < *γ*_*M*_ = ∞, and $Q_{n}\in Q_{n}=\left\{\gamma_{0},\gamma_{1},\ldots ,\gamma_{M-1}\right\}$ is set to *γ*_*m*_ if the channel quality of link *n* is within the range [*γ*_*m*_, *γ*_*m*+1_). In the FSMC, because the link quality is modeled as a random variable with an exponential distribution, the probability density function of the channel quality of link *n* is presented as $f_{Q_{n}}\left(\gamma \right)=\frac{1}{\nu_{n}}\exp\left\{-\frac{\gamma }{\nu_{n}}\right\}$, where *ν*_*n*_ is the mean channel quality of link *n*. Based on *f*_*Q*_*n*__(*γ*), the steady-state probability that *Q*_*n*_ equals a pre-determined link quality *γ*_*m*_ is given as $p_{m}=\left[Q_{n}=\gamma_{m}\right]=\int_{\gamma_{m}}^{\gamma_{m+1}}f_{Q_{n}}\left(\gamma \right)\;d\gamma =\exp\left\{-\frac{\gamma_{m}}{\nu_{n}}\right\}\;-\;\exp\left\{-\frac{\gamma_{m+1}}{\nu_{n}}\right\}$. Then, we denote the transition probability from the link quality *q*_*n*_ at decision epoch *t* to the link quality ${q_{n}}^{\prime }$ at the next decision epoch *t* + 1 as $P_{Q_{n}}\left[{q_{n}}^{\prime }|q_{n}\right]=\left[Q_{n}\left(t+1\right)={q_{n}}^{\prime }|Q_{n}\left(t\right)=q_{n}\right]$, and it is presented as follows:

$$\begin{array}{ccc} P_{Q_{n}}\left[{q_{n}}^{\prime }|q_{n}\right] & = & \frac{D_{m+1}}{R_{n}\left(q_{n}\right)p_{m}},\;\;\text{if}\;\;\;q_{n}=\gamma_{m},{q_{n}}^{\prime }=\gamma_{m+1},m=0,1,...,M-2\\ & = & \frac{D_{m}}{R_{n}\left(q_{n}\right)p_{m}},\;\;\text{if}\;\;\;q_{n}=\gamma_{m},{q_{n}}^{\prime }=\gamma_{m-1},m=1,2,...,M-1\\ & = & 1-P_{Q_{n}}\left[\gamma_{1}|\gamma_{0}\right],\;\;\text{if}\;\;q_{n}={q_{n}}^{\prime }=\gamma_{0}\\ & = & 1-P_{Q_{n}}\left[\gamma_{M-2}|\gamma_{M-1}\right],\;\;\text{if}\;\;q_{n}={q_{n}}^{\prime }=\gamma_{M-1}\\ & = & 1-P_{Q_{n}}\left[\gamma_{m-1}|\gamma_{m}\right]-P_{Q_{n}}\left[\gamma_{m+1}|\gamma_{m}\right],\;\;\text{if}\;\;q_{n}={q_{n}}^{\prime }=\gamma_{m},\;\;m=1,2,\ldots ,M-2. \end{array}$$ (1)

where *R*_*n*_(*q*_*n*_) is the data rate from link *n* when its link quality is *q*_*n*_, and *D*_*m*_ is the expected number of times per second that the link quality passes downward crossings given level *γ*_*m*_. We define data rate *R*_*n*_(*q*_*n*_) later in this paper. Let *V* be the discrete random variable representing the MT’s velocity, and we also define it as a Markov chain later in this paper. Then, we determine *D*_*m*_ as $D_{m}\;=\;\sum_{v}\sqrt{\frac{2\pi \gamma_{m}}{v}}f_{D}\left(v\right)\exp\left\{-\frac{\gamma_{m}}{v}\right\}\left[V=v\right],$ where *f*_*D*_(*v*) is the maximum Doppler frequency when the MT moves at speed *v*. The Doppler frequency is determined as *f*_*D*_(*v*) = *v*/*w*, where *w* is the wavelength of radio communication signals.

### Traffic & velocity model

An active MT can be represented by a traffic flow consisting of *K* + 1 frames. After finishing the transmission of *K* + 1 frames, the active MT leaves the system before becoming active again. The handover decision and corresponding handover execution is performed before the start of each frame after the first frame, such that there exist total *K* handover decision epochs for each active MT. In addition, a traffic flow can be classified into non-real-time and real-time traffic. Compared to non-real-time traffic, real-time traffic is sensitive to delays because of the interactive services being provided. Thus, we set different transmission delay requirements to reflect the characteristics of non-real-time and real-time traffic. Because the time interval between two handover decision epochs is too short, there is little change in the MT’s velocity. Thus, the MT’s velocity is likely to have a correlation with its past and current velocity. To reflect such a characteristic in terms of the velocity, we represent MT’s velocity *V* by adopting a discrete Gauss-Markov mobility model [13], which is widely used to model the MT movement in a cellular network because it captures the essence of the correlation of MT’s velocity in time. In this model, we assumed that MT’s velocity is correlated in time, and that it can be modeled by a discrete Gauss-Markov random process. Let *V*(*t*) be MT’s velocity at the handover decision epoch *t*. Based on the Gauss-Markov mobility model, the velocity can be determined by the recursive realization as follows:

$$\begin{array}{c} V\left(t\right)=\alpha V\left(t-1\right)+\left(1-\alpha \right)\mu +\sigma \sqrt{1-\alpha^{2}}\phi , \end{array}$$ (2)

where *α* ∈ [0, 1] is the memory level, *μ* and *σ* are the mean and standard deviation of *V*, and *ϕ* is an uncorrelated Gaussian process, which is independent of *V*, with zero mean and a unit variance. We define $P_{V}\left[v^{\prime }|v\right]=P\left[V\left(t+1\right)=v^{\prime }|V\left(t\right)=v\right]$ to represent the transition probability from speed *v* at decision epoch *t* to speed *v*′ at the next decision epoch *t*′. By varying *v* and counting the number of different outcomes of *v*′ from Eq (2), we can calculate the transition probability $P_{V}\left[v^{\prime }|v\right]$ by performing simulations. In addition, we can also obtain the steady-state probability (or probability mass function) of MT’s velocity by using the balance equation [14].

## Problem formulation

We determined the validity of the proposed handover strategy using an analytic exploration of the energy-saving potential for optimization in terms of the energy consumption at BSs in HetNets. To do this, we formulated a handover decision problem as a CMDP. In the CMDP, the objective is to minimize the energy consumption generated at BSs during the service time of a traffic flow, and the constraint is to meet the QoS requirements of an MT given in terms of the transmission delay and call-dropping probability.

In general, a CMDP is defined as tuple (*S*, *A*, *T*, *r*, *c*, *d*), where *S* is the state space, *A* is the action space, *T* is the state-transition probability matrix, *e* is the function that reflects the energy consumption, *c* is the function that reflects the call-blocking probability, and *d* is the function that reflects the transmission delay.

State *s* ∈ *S* of an MT includes information on its serving BS, velocity, and link qualities to all available BSs, such that the state space is presented as

$$\begin{array}{c} S=N\times V\times Q_{1}\times Q_{2}\times ...\times Q_{\;|N|}, \end{array}$$ (3)

where × is the Cartesian product operator.

Action space *A* includes BSs that the MT can access at each decision epoch. Action *a* = *n* shows that the MT connects to BS *n* to transmit a frame at the next decision epoch. If the target BS is different from the serving BS, a handover occurs; otherwise, there is no handover, which means that the MT remains connected to the current serving BS.

After taking action *a*(*t*) in state *s*(*t*) at decision epoch *t*, the transition probability to new state *s*′ can be computed as

$$\begin{array}{c} T\left[s^{\prime }|s,a\right]=P\left[s\left(t+1\right)=s^{\prime }|s\left(t\right),a\left(t\right)\right]=\{\begin{array}{cc} P_{V}\left[v^{\prime }|v\right]\prod_{n\in N}P_{Q_{n}}\left[{q_{n}}^{\prime }|q_{n}\right], & \text{if}\;n^{\prime }=a,\\ 0, & \text{otherwise,} \end{array} \end{array}$$ (4)

where it is determined that the action only affects the set of reachable states from the current state.

To measure the energy consumption by BSs to transmit a frame, we define *e*_*f*_(*s*, *a*), which quantifies the amount of energy required to transmit a frame when taking action *a* in state *s*, and it is the sum of transmission cost *e*_*TX*_(*s*, *a*) and handover cost *e*_*HO*_(*s*, *a*) as follows:

$$\begin{array}{c} e_{f}\left(s,a\right)\;=\;e_{TX}\left(s,a\right)\;+\;e_{HO}\left(s,a\right). \end{array}$$ (5)

Transmission cost *e*_*TX*_(*s*, *a*) reflects not only the energy consumed during the transmission of a frame, but also the energy consumed by an electrical circuit that is independent of the transmission power, as given by

$$\begin{array}{c} e_{TX}\left(s,a\right)=\frac{FL}{R_{a}\left(q\right)}\left(P_{a}+P_{a,c}\right), \end{array}$$ (6)

where *P*_*a*_ is the transmission power from BS *a* to the MT, *P*_*a*,*c*_ is the signal processing and electrical circuit power of BS *a*, and *FL* is the number of bits in a frame. In addition, $R_{a}\left(q\right)$ is the data rate achieved between BS *a* and the MT when the link qualities from accessible BSs to the MT are given as a set of $q=[q_{1},...,q_{a},...q_{\left|N\right|}]$, and it is expressed as

$$\begin{array}{c} R_{n}\left(q|U_{n}=m\right)=\frac{BW}{m+1}\log\left(1+SINR_{n}\left(q\right)\right), \end{array}$$ (7)

where *U*_*n*_ is the number of MTs connected to the BS, and *BW* is the available bandwidth. In Eq (7), *BW*/(*m* + 1) refers to the bandwidth per connection, which is based on an assumption that the available bandwidth at each BS is equally shared among all connected MTs. Note that unlike a link quality is modeled as a stochastic process, e.g., FSMC, there are few studies that model the variation in the number of MTs connected to each BS according to the passage of time as a stochastic process; instead, we treat *U*_*n*_ as a random variable. Let us consider HetNets, where there is a large number of MTs, each of which has a small probability of being active. For such a condition, as the number of attached MTs per BS increases, its distribution is best approximated by the Poisson distribution [15]. Therefore, we consider *U*_*n*_ to be Poisson distributed with parameter λ_*n*_, where λ_*n*_ is the MT’s density within the coverage area of BS *n*. Subsequently, for established link *n*, its unconditional achievable date rate $R_{n}\left(q\right)$ can be determined as

$$\begin{array}{ccc} R_{n}\left(q\right) & = & \sum_{m=0}^{\infty }R_{n}\left(q|U_{n}=m\right)\mathrm{Pr}\left[U_{n}=m\right]\\ & = & \frac{W\log_{2}\left(1+SINR_{n}\left(q\right)\right)}{\lambda_{n}}e^{-\lambda_{n}}\sum_{m=0}^{\infty }\frac{{\lambda_{n}}^{m+1}}{(m+1)!}\\ & = & \frac{W\log_{2}\left(1+SINR_{n}\left(q\right)\right)}{\lambda_{n}}e^{-\lambda_{n}}\left[\left(\sum_{m+1=0}^{\infty }\frac{{\lambda_{n}}^{m+1}}{(m+1)!}\right)-\frac{{\lambda_{n}}^{-1+1}}{\left(-1+1\right)}\right]\\ & = & \frac{W\log_{2}\left(1+SINR_{n}\left(q\right)\right)}{\lambda_{n}}\left(1-e^{-\lambda_{n}}\right). \end{array}$$ (8)

By using Eq (8), *e*_*TX*_(*s*, *a*) can be rewritten as follows:

$$\begin{array}{c} e_{TX}\left(s,a\right)=\frac{FL\lambda_{a}\left(P_{a}+P_{a,c}\right)}{BW\log\left(1+SINR_{a}\left(q\right)\right)\left(1-e^{-\lambda_{a}}\right)}. \end{array}$$ (9)

For serving and target BSs, the handover cost *e*_*HO*_(*s*, *a*) captures the energy consumption that is incurred by signaling exchanges and processing loads during the handover-execution phase. This value can vary according to the network type of the serving and target BSs [16]. In this work, it is assumed to be static owing to the limitation of not having the exact measurement over various handover-execution phases, as given below:

$$\begin{array}{c} e_{HO}\left(s,a\right)=\{\begin{array}{cc} e_{HO}^{i,a}, & \text{if}\;i\neq a,\\ 0, & \text{if}\;i=a, \end{array} \end{array}$$ (10)

where $e_{HO}^{i,a}$ is the energy consumed when switching the MT from the serving BS *i* to the target BS *a*.

Given policy *π* and the number of frames in a traffic flow *K* + 1, the expected total energy consumption when serving the MT over the lifetime of its connection from initial state *s* can be expressed as:

$$\begin{array}{c} E^{\pi }\left(s\right)=\Phi_{s}^{\pi }\left[\Phi_{K}\left\{\sum_{t=1}^{K}\delta^{t-1}e_{f}\left(s\left(t\right),a\left(t\right)\right)\right\}\right], \end{array}$$ (11)

where $\Phi_{s}^{\pi }\left[\cdot \right]$ denotes the expectation under policy *π* and initial state *s*, *δ* ∈ [0, 1) denotes the discount factor, which determines the importance of the future value at the current decision epoch, and *e*_*f*_(*s*(*t*), *a*(*t*)) is the energy consumption at decision epoch *t*. In addition, Φ_*K*_[⋅] denotes the expectation under random variable *K*. Because different MTs may have connections with different numbers of frames to be transmitted, the number of handover decision epochs *K* is assumed to be a discrete random variable with a Geometric distribution having mean 1/(1 − λ), such that *E*^*π*^(*s*) in Eq (11) can be rewritten as follows:

$$\begin{array}{ccc} E^{\pi }\left(s\right) & = & \Phi_{s}^{\pi }\left[\sum_{k=1}^{\infty }\sum_{t=1}^{k}\delta^{t-1}e_{f}\left(s\left(t\right),a\left(t\right)\right)\lambda^{k-1}\left(1-\lambda \right)\right]\\ & = & \Phi_{s}^{\pi }\left[\sum_{t=1}^{\infty }\delta^{t-1}e_{f}\left(s\left(t\right),a\left(t\right)\right)\left(1-\lambda \right)\sum_{k=t}^{\infty }\lambda^{k-1}\right]\\ & = & \Phi_{s}^{\pi }\left[\sum_{t=1}^{\infty }\delta^{t-1}\lambda^{t-1}e_{f}\left(s\left(t\right),a\left(t\right)\right)\right]\\ & = & \Phi_{s}^{\pi }\left[\sum_{t=1}^{\infty }\varsigma^{t-1}e_{f}\left(s\left(t\right),a\left(t\right)\right)\right], \end{array}$$ (12)

where ς=δλ∈[0,1) is the discount factor for the random number of decision epochs per traffic flow.

To complete the description of the CMDP, we require details regarding the remaining two functions that track the QoSs of the MT. First, we define *d*(*s*, *a*), which captures the delay to transmit a frame, and it is presented as

$$\begin{array}{c} d\left(s,a\right)=\frac{FL}{R_{a}\left(q\right)}. \end{array}$$ (13)

Based on Eq (13), the expected total transmission delay over the lifetime of a connection is expressed as

$$\begin{array}{ccc} D^{\pi }\left(s\right) & = & \Phi_{s}^{\pi }\left[E_{K}\left\{\sum_{t=1}^{K}\delta^{t-1}d\left(s\left(t\right),a\left(t\right)\right)\right\}\right]\\ & = & \Phi_{s}^{\pi }\left[\sum_{t=1}^{\infty }\delta^{t-1}\lambda^{t-1}d\left(s\left(t\right),a\left(t\right)\right)\right]\\ & = & \Phi_{s}^{\pi }\left[\sum_{t=1}^{\infty }\varsigma^{t-1}d\left(s\left(t\right),a\left(t\right)\right)\right]. \end{array}$$ (14)

Note that real-time traffic is more sensitive than non-real-time traffic in terms of the delay, such that different delay constraints are needed to differentiate between them. For non-real-time traffic, the expected total transmission delay Eq (14) should be less than the total delay threshold $D_{total}^{th}$ as follows:

$$\begin{array}{c} \Phi_{s}^{\pi }\left[\sum_{t=1}^{\infty }\varsigma^{t-1}d\left(s\left(t\right),a\left(t\right)\right)\right]\leq D_{total}^{th}. \end{array}$$ (15)

For non-real-time traffic, it is acceptable to consider the total transmission delay because the main concern when transmitting non-real-time traffic is the length of time for which it should wait to transmit all of the data. For real-time traffic, we consider an additional constraint at each handover decision epoch. That is, the transmission delay to service the current frame, i.e., *d*(*s*(*t*), *a*(*t*)), should be less than the frame delay threshold $D_{frame}^{th}$, as follows:

$$\begin{array}{c} d\left(s\left(t\right),a\left(t\right)\right)\leq D_{frame}^{th}. \end{array}$$ (16)

A call may be dropped for various reasons, such as the MT’s velocity, insufficient radio resources, and the presence of dead zones [17]. Of the different reasons, we focus on the MT’s velocity. When an MT’s speed increases, the call-dropping probability during the handover execution increases. Thus, we define *c*(*s*, *a*) in order to capture the call-dropping probability when action *a* is taken in state *s*, and it is expressed as

$$\begin{array}{c} c\left(s,a\right)=\{\begin{array}{cc} 0, & \text{if}\;\;i=a,\\ 0, & \text{if}\;i\neq a,\;\;\;0\leq V<V_{\min}\\ \frac{\left(V-V_{\min}\right)}{\left(V_{\max}-V_{\min}\right)}, & \text{if}\;\;i\neq a,\;\;\;V_{\min}\leq V<V_{\max}\\ 1, & \text{if}\;i\neq a,\;\;\;V\geq V_{\max} \end{array} \end{array}$$ (17)

where *V*_min_ and *V*_max_ are the minimum and maximum velocity thresholds, respectively. Some MTs may be handed over to a target BS in order to achieve a better data rate although there is a risk that the connection may be dropped during the handover execution. On the other hand, others may be fearful of switching to the target BS. Based on Eq (17), the expected average call-dropping probability over the lifetime of a connection can be determined as

$$\begin{array}{ccc} C^{\pi }\left(s\right) & = & \left(1-\delta \right)\Phi_{s}^{\pi }\left[\Phi_{k}\left\{\sum_{t=1}^{k}\delta^{k-1}c\left(s\left(t\right),a\left(t\right)\right)\right\}\right]\\ & = & \left(1-\delta \right)\Phi_{s}^{\pi }\left[\sum_{t=1}^{\infty }\delta^{t-1}\lambda^{t-1}c\left(s\left(t\right),a\left(t\right)\right)\right]\\ & = & \Phi_{s}^{\pi }\left[\sum_{t=1}^{\infty }\varsigma^{t-1}\left(1-\delta \right)c\left(s\left(t\right),a\left(t\right)\right)\right], \end{array}$$ (18)

where *C*^*π*^(*s*) is the expected total call-dropping probability multiplied by a normalizing constant (1 − *δ*). From [16], we note that by using the normalizing constant, the expected total value will converge to the expected average value when we use stationary policies. Because we consider only stationary policies, *C*^*π*^(*s*) is the expected average call-dropping probability, and its value must not exceed the call-dropping threshold $C_{total}^{th}$, as follows:

$$\begin{array}{c} C^{\pi }\left(s\right)\leq C_{total}^{th}. \end{array}$$ (19)

Finally, we have described all of the components of a CMDP. Based on the description, we can describe optimization problems in obtaining an energy-efficient handover strategy. Let Π be the set of all feasible policies. For non-real-time traffic, we can formulate an optimization problem as

$$\begin{array}{ccc} \underset{\pi \in \Pi }{\text{min}}\;E^{\pi }\left(s\right) & = & \Phi_{s}^{\pi }\left[\sum_{t=1}^{\infty }\varsigma^{t-1}r\left(s(t),a(t)\right)\right]\\ \text{s. t.}\;\;\;C_{}^{\pi }\left(s\right) & = & \Phi_{s}^{\pi }\left[\sum_{t=1}^{\infty }\varsigma^{t-1}\left(1-\delta \right)c\left(s(t),a(t)\right)\right]\leq C_{total}^{th}\\ D^{\pi }\left(s\right) & = & \Phi_{s}^{\pi }\left[\sum_{t=1}^{\infty }\varsigma^{t-1}d\left(s_{t},a_{t}\right)\right]\leq D_{total}^{th}. \end{array}$$ (20)

For real-time traffic, we can also formulate an optimization problem as

$$\begin{array}{ccc} \underset{\pi \in \Pi }{\text{min}}\;E^{\pi }\left(s\right) & = & \Phi_{s}^{\pi }\left[\sum_{t=1}^{\infty }\varsigma^{t-1}r\left(s\left(t\right),a\left(t\right)\right)\right]\\ \text{s. t.}\;\;\;C_{}^{\pi }\left(s\right) & = & \Phi_{s}^{\pi }\left[\sum_{t=1}^{\infty }\varsigma^{t-1}\left(1-\delta \right)c\left(s\left(t\right),a\left(t\right)\right)\right]\leq C_{total}^{th}\\ D^{\pi }\left(s\right) & = & \Phi_{s}^{\pi }\left[\sum_{t=1}^{\infty }\varsigma^{t-1}d\left(s\left(t\right),a\left(t\right)\right)\right]\leq D_{total}^{th}\\ d(s(t),a(t)) & \leq & D_{frame}^{th}. \end{array}$$ (21)

## Optimal policy for CMDP

To obtain optimal policies for constrained control Problems (21) and (22), we adopt the ideas of dynamic programming. Dynamic programming is a method that is employed to solve a complex problem by breaking it down into simpler subproblems in mathematics, computer science, economics, etc. To obtain the optimal solution of a nonconstrained control problem, dynamic programming such as value iteration, policy iteration, and modified policy iteration algorithms can be applied to it, where a minimization problem over all policies is transformed into a set of minimization problems over a much smaller set of actions.

To utilize the dynamic programming techniques for the constrained problems, we used a standard Lagrangian approach, which transforms a constrained minimization problem into an inf-sup problem of Lagrangian [18] as follows:

$$\begin{array}{c} G\left(s\right)=\underset{\pi \in \Pi }{\inf}\underset{\mu }{\sup}J_{\mu }^{\pi }\left(s\right), \end{array}$$ (22)

where *G*(*s*) represents the value of the constrained problems, and $J_{\mu }^{\pi }\left(s\right)$ denotes the Lagrangian of the constrained problems. The Lagrangian is defined as the sum of the function to be minimized and all of the other functions to be constraints weighted by some constants called Lagrange multipliers as follows:

$$\begin{array}{c} J_{\mu }^{\pi }\left(s\right)=E^{\pi }\left(s\right)+\mu_{1}\left(C^{\pi }\left(s\right)-C_{total}^{th}\right)+\mu_{2}\left(D^{\pi }\left(s\right)-D_{total}^{th}\right), \end{array}$$ (23)

where *μ*_1_ and *μ*_2_ are nonnegative Lagrange multipliers corresponding to constraints. Note that the sup-inf problem is more familiar than the inf-sup problem, as it involves first minimizing with respect to the policies and then maximizing with respect to ***μ***. Thus, we change the order of the inf and the sup in Eq (22) by invoking a saddle-point theorem, such that

$$\begin{array}{c} G\left(s\right)=\underset{\mu }{\sup}\underset{\pi \in \Pi }{\inf}J_{\mu }^{\pi }\left(s\right). \end{array}$$ (24)

In summary, solving a constrained optimization problem is equivalent to solving a nonconstrained sup-inf problem.

For the given ***μ***, our first challenge is to identify the optimal policy π* from among all the feasible policy Π such that the expected total energy consumption is minimized, as given by

$$\begin{array}{c} G_{\mu }\left(s\right)=\underset{\pi \in \Pi }{\inf}J_{\mu }^{\pi }\left(s\right), \end{array}$$ (25)

where *G*_***μ***_(*s*) denotes the minimum expected energy consumption under a given ***μ***. To obtain *G*_***μ***_(*s*), we apply dynamic programming for an unconstrained control problem. From Eq (23), we can define the Lagrangian cost function *j*(*s*, *a*, ***μ***) as follows:

$$\begin{array}{c} j\left(s,a,\mu \right)=e_{f}\left(s,a\right)+\mu_{1}\left(1-\delta \right)c\left(s,a\right)+\mu_{2}d\left(s,a\right). \end{array}$$ (26)

Accordingly, we can represent the Bellman optimality equation as follows:

$$\begin{array}{c} G_{\mu }\left(s\right)=\min_{a\in A_{s}}\left[j\left(s,a,\mu \right)+\varsigma \sum_{s^{\prime }\in S}T(s^{\prime }|s,a)\;G_{\mu }\left(s^{\prime }\right)\right]. \end{array}$$ (27)

In order to obtain the solutions of the optimality equation, we used the value iteration algorithm, described in Algorithm 1, which is a method that can be used to find *ε*-optimal policies for a discounted MDP. The detailed convergence proof of the value iteration algorithm is described in [19].

**Algorithm 1** Value iteration algorithm

1: Set $G_{\mu }^{p}\left(s\right)=0$ for each state *s*, *ε*_1_ > 0, and *p* = 0.

2: For each state *s*, compute $G_{\mu }^{p+1}\left(s\right)$ using

$$\begin{array}{c} G_{\mu }^{p+1}\left(s\right)=\min_{a\in A_{s}}\left[j\left(s,a,\mu \right)+\varsigma \sum_{s^{\prime }\in S}T(s^{\prime }|s,a)\;G_{\mu }^{p}\left(s^{\prime }\right)\right]. \end{array}$$

3: If $∥G_{\mu }^{p+1}\left(s\right)-G_{\mu }^{p}\left(s\right)∥<\frac{\varepsilon_{1}\left(1-\varsigma \right)}{2\varsigma }$, go to step 4. Otherwise, increase *p* by 1 and return to step 2.

4: For each state *s*, obtain the stationary policy using

$$\begin{array}{c} \theta_{\mu }\left(s\right)=\arg\min_{a\in A_{s}}\left[j\left(s,a,\mu \right)+\varsigma \sum_{s^{\prime }\in S}T(s^{\prime }|s,a)\;G_{\mu }^{p+1}\left(s^{\prime }\right)\right], \end{array}$$

and stop.

After finding the stationary policy *θ*_***μ***_(*s*) from Algorithm 1, we performed the update of Lagrange multipliers used to obtain the value *G*(*s*) by solving the equation below:

$$\begin{array}{c} G\left(s\right)=\underset{\mu }{\sup}G_{\mu }\left(s\right)=\underset{\mu_{1}}{\sup}\mu_{1}\left(C^{\theta_{\mu }}\left(s\right)-C_{total}^{th}\right)+\underset{\mu_{2}}{\sup}\mu_{2}\left(D^{\theta_{\mu }}\left(s\right)-D_{total}^{th}\right), \end{array}$$ (28)

where $C^{\theta_{\mu }}\left(s\right)$ and $D^{\theta_{\mu }}\left(s\right)$ are computed as follow:

$$\begin{array}{c} C^{\theta_{\mu }}\left(s\right)\;=c\left(s,\theta_{\mu }\left(s\right)\right)+\varsigma \sum_{s^{\prime }\in S}T(s^{\prime }|s,\theta_{\mu }\left(s\right))\;C^{\theta_{\mu }}\left(s^{\prime }\right), \end{array}$$ (29)

$$\begin{array}{c} D^{\theta_{\mu }}\left(s\right)\;=d\left(s,\theta_{\mu }\left(s\right)\right)+\varsigma \sum_{s^{\prime }\in S}T(s^{\prime }|s,\theta_{\mu }\left(s\right))\;D^{\theta_{\mu }}\left(s^{\prime }\right). \end{array}$$ (30)

To update $\mu_{1}^{z+1}$ and $\mu_{2}^{z+1}$, we can apply a gradient-decent method as follows:

$$\begin{array}{ccc} \mu_{1}^{z+1} & = & \mu_{1}^{z}+\frac{1}{\tau_{1}}\left(C^{\theta_{\mu }^{z}}\left(s\right)-C_{total}^{th}\right), \end{array}$$ (31)

$$\begin{array}{ccc} \mu_{2}^{z+1} & = & \mu_{2}^{z}+\frac{1}{\tau_{2}}\left(D^{\theta_{\mu }^{z}}\left(s\right)-D_{total}^{th}\right), \end{array}$$ (32)

where *z* is the iteration number. In addition, *τ*_1_ and *τ*_2_ are constant step sizes that must be sufficiently small to ensure convergence to optimal solutions. Algorithm 2 describes the procedure employed to achieve an optimal solution of CMDP.

**Algorithm 2** Procedure to obtain an optimal solution of CMDP

1: Initialize $\mu_{1}^{1}$ and $\mu_{2}^{1}$ as an arbitrary positive number, and set *z* = 1 and *ε*_2_ > 0.

2: For each state *s*, obtain $\theta_{\mu }^{z}\left(s\right)$ using Algorithm 1.

3: For each state *s*, compute $C^{\theta_{\mu }^{z}}\left(s\right)$ and $D^{\theta_{\mu }^{z}}\left(s\right)$ using

$$\begin{array}{ccc} C^{\theta_{\mu }^{z}}\left(s\right) & = & c\left(s,\theta_{\mu }^{z}\left(s\right)\right)+\varsigma \sum_{s^{\prime }\in S}T(s^{\prime }|s,\theta_{\mu }^{z}\left(s\right))\;C^{\theta_{\mu }^{z}}\left(s^{\prime }\right),\\ D^{\theta_{\mu }^{z}}\left(s\right) & = & d\left(s,\theta_{\mu }^{z}\left(s\right)\right)+\varsigma \sum_{s^{\prime }\in S}T(s^{\prime }|s,\theta_{\mu }^{z}\left(s\right))\;D^{\theta_{\mu }^{z}}\left(s^{\prime }\right). \end{array}$$

4: Update the Lagrange multipliers using

$$\begin{array}{ccc} \mu_{1}^{z+1} & = & \mu_{1}^{z}+\frac{1}{\tau_{1}}\left(C^{\theta_{\mu }^{z}}\left(s\right)-C_{\text{total},\text{th}}\right),\\ \mu_{2}^{z+1} & = & \mu_{2}^{z}+\frac{1}{\tau_{2}}\left(D^{\theta_{\mu }^{z}}\left(s\right)-D_{\text{total},\text{th}}\right). \end{array}$$

5: If $∥\mu_{1}^{z+1}-\mu_{1}^{z}∥<\varepsilon_{2}$ and $∥\mu_{2}^{z+1}-\mu_{2}^{z}∥<\varepsilon_{2}$, stop. Otherwise, increase *z* by 1 and return to step 2.

## Discussion on practical implementation of proposed handover strategy

When implementing the proposed handover strategy in a practical wireless system, a computational complexity issue with respect to obtaining an optimal policy of CDMP may arise. To reduce the computational complexity, we can consider several alternatives. For example, one is to make a solution table including the optimal policies of CMDP with respect to all possible states that an MT may have in advance using a scheduler at the macro BS, which is adopted in many existing works [10] [20]. The other is to utilize a finite amount of information on the stochastic behavior of handover parameters for a handover decision. To do so, we should model an optimization problem as a finite-horizon CDMP. However, this may decrease the amount of energy savings realized compared to the opposite case (i.e., an infinite amount of information on the stochastic behavior of link qualities is utilized for a handover decision).

## Simulation results

To obtain the simulation results, we considered HetNets in which an MT is located within the overlapping area made by two BSs belonging to different types of network, and where the MT can access both BSs during the lifetime of a traffic flow. This setting follows the commonly adopted macro-femto framework, and it is easily extended to a more complex one. Of the different parameters, we set the nominal values as follows: *FL* = 500000 symbols, *BW* = 10 MHz, *P*_1_ = 10 W, *P*_1_ = 6 W, *P*_1,*c*_ = *P*_2,*c*_ = 2 W, *q*_1_ ∈ {0, 6, 10, 14, ∞} dB, *q*_2_ ∈ {0, 6, 8, 12, ∞} dB, *ν*_1_ = 6 dB, *ν*_2_ = 8 dB, λ_1_ = 1, λ_2_ = 0.6. In addition, the velocity of the quantized MT is 6, 14, and 22 km/h, and the maximum and minimum velocity thresholds are 6 and 30 km/h, respectively. For the Gauss-Markov model, the memory level is 0.5, and the mean and standard deviation of the MT’s velocity are 6 and 1 km/h, respectively. To find the optimal solution of CMDP, we use the Markov Decision Processes Toolbox in MATLAB. Because it is basically for MDP, we modified the m-file in Toolbox to enable its use for CMDP.

We compared the performance of the proposed vertical handover (VHO) strategy to that of the SINR-based VHO strategy [21], Rate-based VHO strategy [22], and NO-VHO strategy [10]. In each decision epoch, the SINR-based VHO strategy utilizes SINR values from among accessible BSs for a handover decision. In contrast, the rate-based VHO strategy utilizes achievable data rates of the BSs. In the NO-VHO strategy, there is no VHO during the service time of a traffic flow, which implies that the MT is connected to only one BS during the service time of a traffic flow. The performance metrics considered are the expected total energy consumption and the total number of handovers. The expected total energy consumption has been defined in Eq (11), while the total number of handovers refers to the total handover count over the entire service time of a traffic flow for all possible initial states.

**Fig 2:**
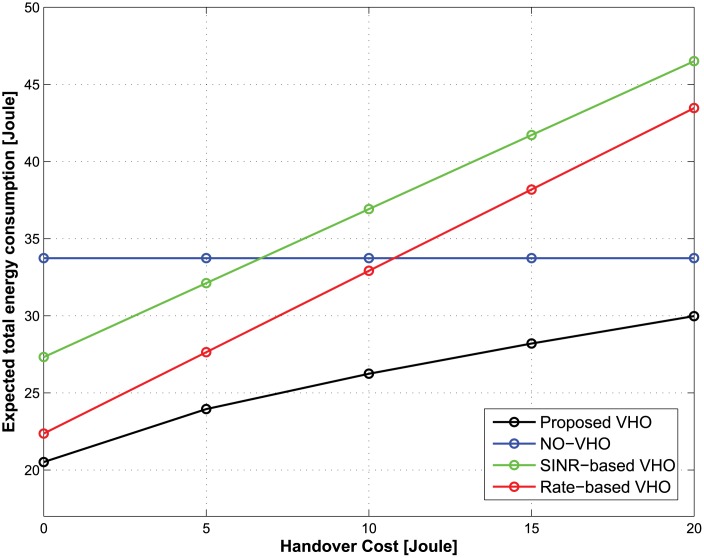
Expected total energy consumption under different handover costs.

**Fig 3:**
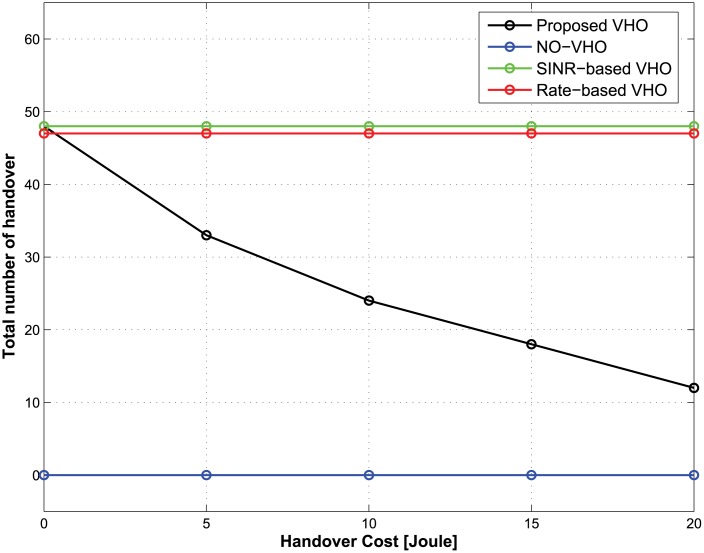
Total number of handovers under different handover costs.

**The case of nonreal-time traffic:** Figs 2 and 3 show the expected total energy consumption and the total number of handovers during the lifetime of a traffic flow with respect to the change in handover costs. The total delay threshold, the average call-dropping probability threshold, and the discount factor of the CMDP framework are set to be 1.2 s, 0.08%, and 0.9, respectively. Fig 2 shows that the optimal policy from the proposed strategy offers the lowest expected total energy consumption compared with other strategies regardless of the handover costs. Fig 3 shows that in the case of the proposed strategy, as the handover cost increases, the total number of handovers decreases. From an energy-saving perspective, this implies that although the achievable data rate from the current BS is relatively lower than other candidate BSs, maintaining the current BS under the condition that the QoS constraints are satisfied may be better strategy than performing a handover to other candidate BSs. This is because each handover incurs some energy consumption, which may be greater than the small energy savings realized from the handover. That is, the proposed strategy can accurately identify the diminishing potential of energy saving at increasing handover costs. On the other hand, SINR-based and rate-based strategies select a BS depending only on the SINR and the achievable rate, and no handover takes place in the NO-VHO strategy. As a result, the total number of handovers for those strategies is constant regardless of the handover cost. Because the objective of our work is to minimize the energy consumption while preserving the QoS experienced by MTs, we conclude that the proposed strategy outperforms other strategies from the perspective of energy efficiency.

**Fig 4:**
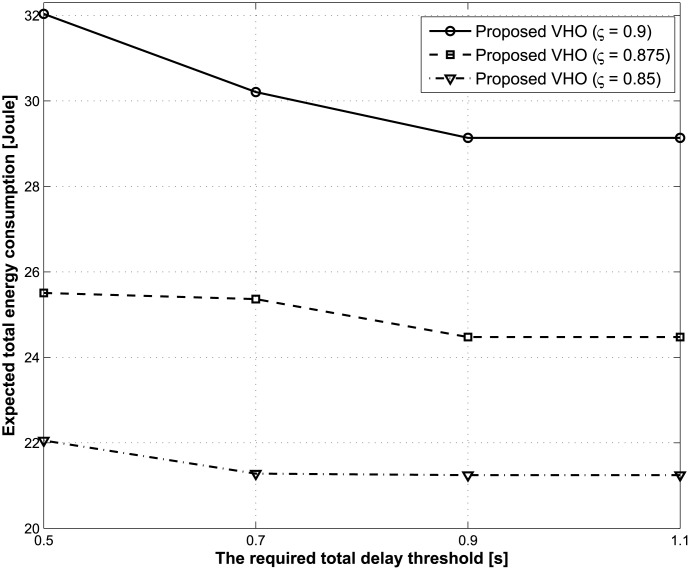
Expected total energy consumption under different required total delay thresholds.

**Fig 5:**
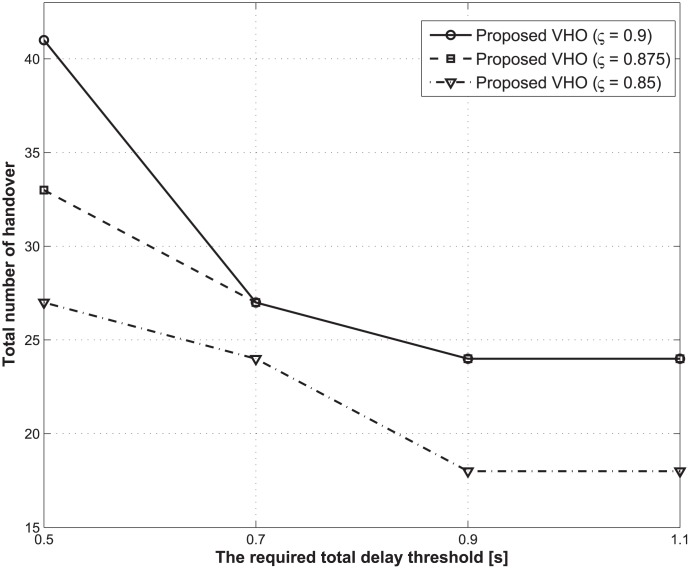
Total number of handovers under different required total delay thresholds.

Figs 4 and 5 illustrate the expected total energy consumption and the total number of handovers during the lifetime of a traffic flow with respect to the change in the required total delay thresholds. In this case, the average call-dropping probability threshold is fixed at 0.08%. From Figs 4 and 5, we see that when the required total delay threshold increases, both the expected total energy consumption and the total number of handovers decrease regardless of the values for the discount factor. As previously mentioned, the main advantage of the proposed strategy is to achieve energy saving by ensuring that the current BS does not hand over to other BSs frequently under the condition of preserving the total delay constraint. However, as the total delay constraint gradually becomes smaller (i.e., the required total delay threshold decreases), the number of cases for which a handover should inevitably occur increase in order to guarantee the total delay constraint, which results in additional energy consumption.

**Fig 6:**
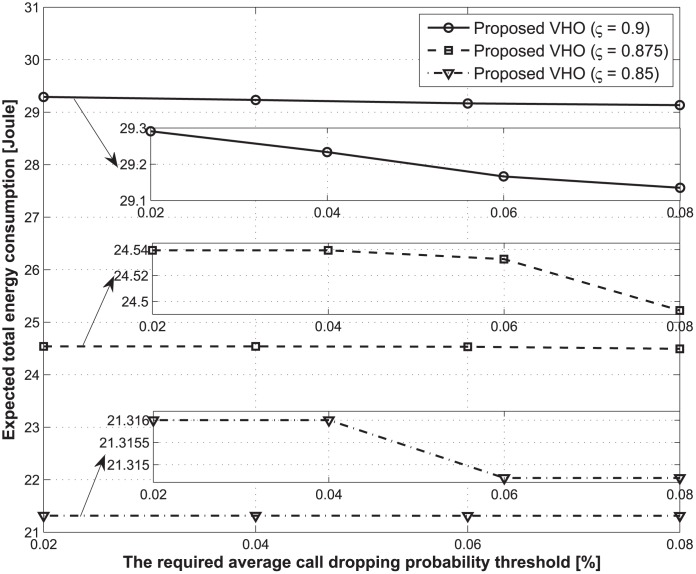
Expected total energy consumptions during the lifetime of a traffic flow for different required average call-dropping probability thresholds.

**Fig 7:**
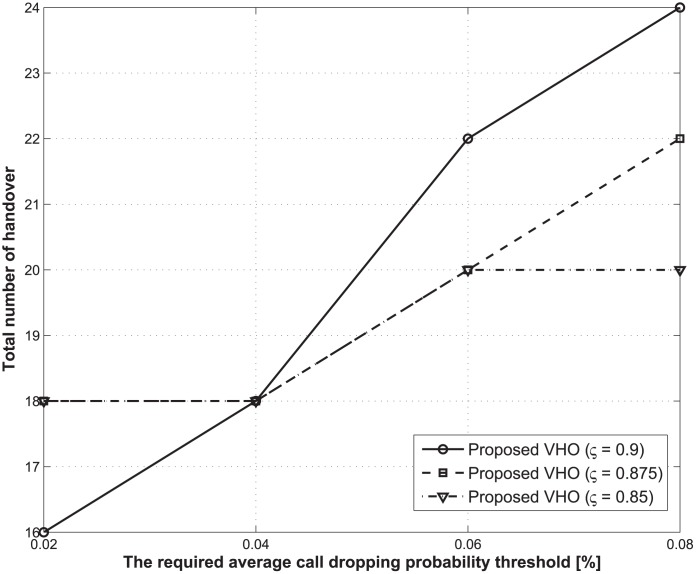
Total number of handovers during the lifetime of a traffic flow under different required average call dropping probability thresholds.

Figs 6 and 7 present the expected total energy consumption and the total number of handovers over the lifetime of a traffic flow with respect to changes in the required average call-dropping probability thresholds. In this case, the total delay threshold is fixed at 1.2 s. From Figs 6 and 7, we also showed that as the required average call-dropping probability threshold increases, the expected total energy consumption decreases, whereas the total number of handovers increases regardless of the values of the discount factor. To analyze these results, we focused on the case where an MT moves to the coverage area of BS 2 from that of BS 1. In this case, although the MT can obtain a higher data rate from BS 2 when compared with BS 1 in order for a handover to occur from the perspective of energy saving, the handover may not be performed. This is because of the risk of call dropping, which is caused by the high velocity of the MT, and which also results in additional energy consumption. This tendency will become more frequent as the expected average call-dropping constraint becomes tighter.

**Fig 8:**
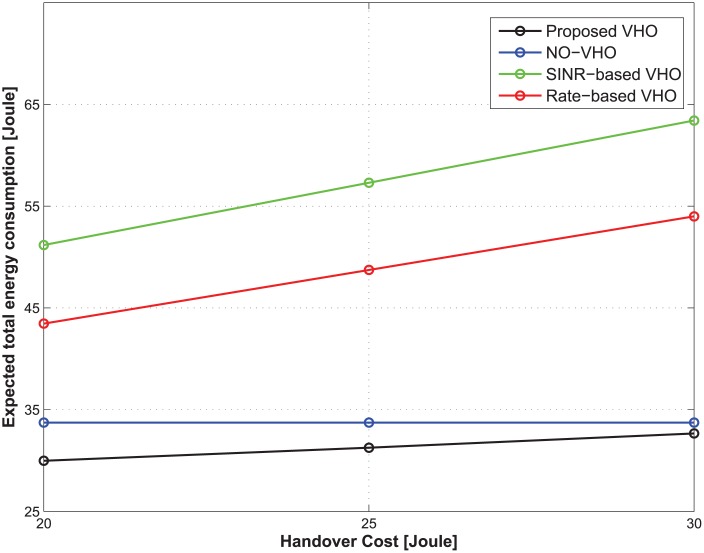
Expected total energy consumption under different handover costs.

**Fig 9:**
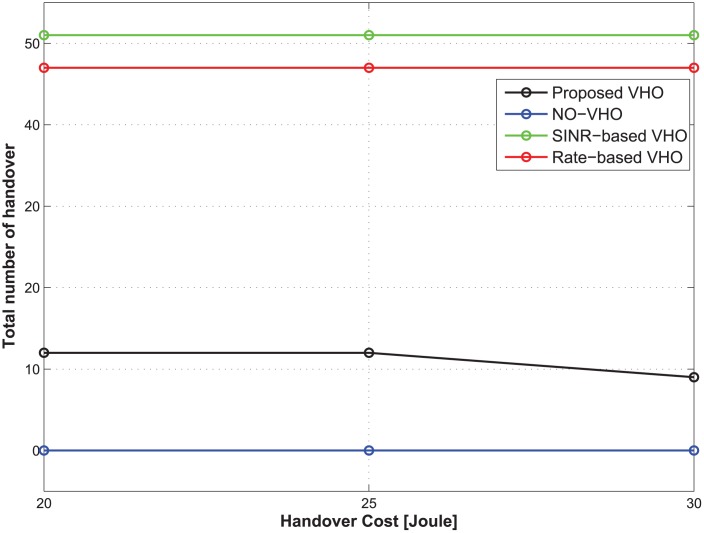
Total number of handovers for different handover costs.

**In the case of real-time traffic:** Figs 8 and 9 illustrate the expected total energy consumption and the total number of handovers over the lifetime of a traffic flow with respect to the change in handover costs. We set the total delay threshold, frame delay threshold, average call-dropping probability threshold, and discount factor of the MDP framework to 1.2 s, 0.14 s, 0.08%, and 0.9, respectively. For all strategies, we assumed that when an MT can access only one BS, it connects to the BS regardless of the frame delay threshold in order to prevent the disconnection of a call. Fig 9 shows that, similar to the case of nonreal-time traffic, the proposed strategy outperforms other strategies in terms of energy saving. In addition, with respect to the high handover cost, the NO-VHO strategy may also be a good alternative compared to the rate-based and SINR-based strategies. From Fig 9, we also observed that in the proposed strategy, the total number of handovers does not change at the handover costs of 20 and 25, which implies that at the handover cost of 25, handover occurrences are forced because of the frame delay threshold, even though those handovers do not contribute to energy savings.

**Fig 10:**
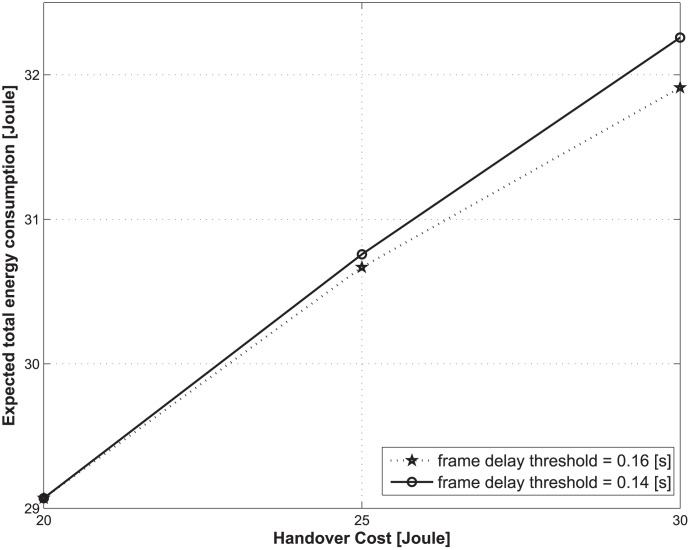
Expected total energy consumption under different required frame delay thresholds.

Fig 10 shows the expected total energy consumption over the lifetime of a traffic flow with respect to the change in handover costs and frame delay thresholds. As shown in the figure, as the frame delay threshold decreases, the expected total energy consumption increases. Note that for the same handover cost, the action set may be different according to the frame delay threshold. As the frame delay threshold decreases, the number of available actions decreases, which can also lead to an inefficient network operation from the perspective of energy saving.

**Fig 11:**
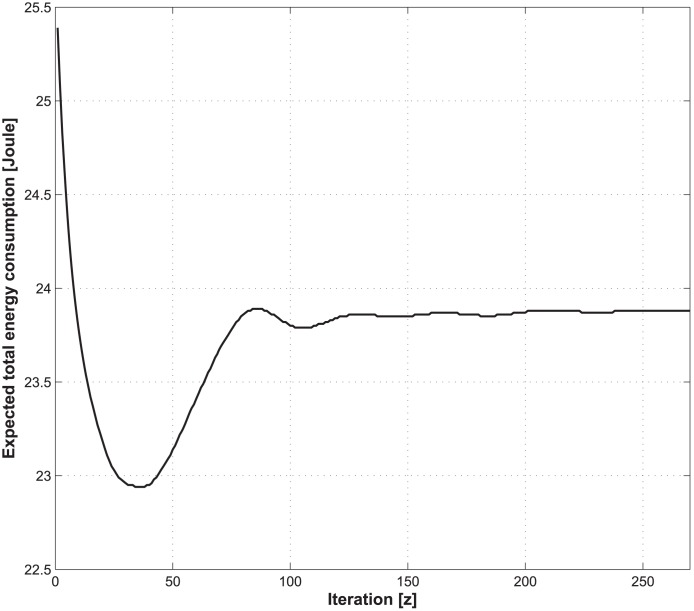
Illustration of the convergence of the expected total energy consumption.

**Fig 12:**
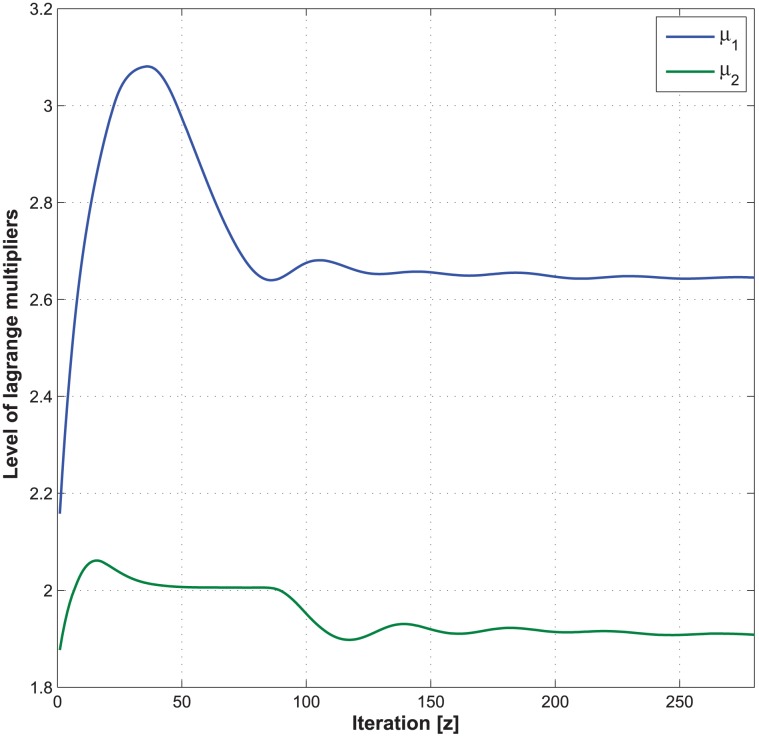
Illustration of the convergence of Lagrange multipliers.

Figs 11 and 12 illustrate the convergence of the expected total energy consumption and Lagrange multipliers, given that the handover cost, the total delay threshold, the average call-dropping probability threshold, and the discount factor of CMDP framework are set to be 5, 1.2 s, 0.08%, and 0.9, respectively.

## Conclusion

To improve the energy efficiency in HetNets, we proposed a handover strategy. The objective of this study was to realize energy savings at BSs while serving a traffic flow, without compromising QoS requirements in terms of the transmission delay and call-dropping probability. The proposed strategy was based on a CMDP that captures not only the current system state, but also stochastic behaviors of handover parameters for a handover decision, and it can be applied to both nonreal-time and real-time calls by differentiating between the delay constraints. Simulation results showed that the proposed handover strategy can reduce the energy consumption by at least 12% for nonreal-time traffic and 3% for real-time traffic compared with the existing handover strategies. In addition, depending on the changes in the QoS requirements for the traffic flow, the performance of the proposed strategy will vary depending on the characteristics of the QoS factors.
